# Strengthening health systems through surgery

**DOI:** 10.1136/bmjgh-2024-017782

**Published:** 2024-11-07

**Authors:** Dion G Morton, Abdul Ghaffar

**Affiliations:** 1 Clinical and Experimental Medicine, University of Birmingham, Birmingham, UK; 2 Department of Community Health Sciences, The Aga Khan University, Karachi, Pakistan

**Keywords:** Global health, Health policies and all other topics, Health economics

 Surgical teams, including managers, nurses, anaesthetists and surgeons, play a pivotal role in delivering essential health services, strengthening the wider health systems, especially in the face of external threats from pandemics, extremes of weather or war. This can be achieved by strengthening care pathways from the community for non-communicable diseases (NCD) building resilient operating systems that strengthen the whole hospital, and introducing low-carbon, cost-efficient care systems. A key area for system strengthening and safe expansion of services is primary healthcare (PHC). There is good evidence that linking PHC facilities to the (first referral) hospitals improves the health outcomes.^1^ However, primary research evidence linking community and PHC facilities to surgical care is still lacking and is required to justify service expansion. Because surgery is easily measured and outcomes directly accountable, surgical teams are ideally placed to evaluate the impact of such service changes on the hospital system and the individual patients they care for.

All advances in healthcare require high-quality evidence to justify investment of public funds. But nowhere is this more apparent than in the development of surgical services. These are complex systems that require substantial investment in infrastructure, specialist training for surgeons, anaesthetists and theatre staff, as well as effective pathways of referral and post-surgery rehabilitation. The development of surgical care across the world is lagging behind that of other essential services, such as maternal and child health, malaria, tuberculosis and NCDs. This may be explained by the significant initial investment required. Kamarajah and colleagues argue, in the accompanying article,^2^ that this lack of prioritisation may also be due to a failure of integration of surgery into health systems and the absence of robust evidence supporting the principle that surgical service development will strengthen the health system and improve the health outcomes. Pathways of care are essential to safe surgery. Delay in care leads to emergency presentation and adverse outcomes including death,^3^ even for simple conditions such as appendicitis^4^ or inguinal hernia.^5^ These pathways can strengthen other components of the health system through training for community health workers, improving referral for emergency medical conditions and elective management of NCDs.^6^ The expanding surgical and perioperative research networks across the low- and middle-income countries (LMICs) could be better used, especially by the health system community, given their capability and promise to develop the evidence base and evaluate the impact of change both on the health system and on the individual patients.

In recent years, surgical teams, a powerful and respected group within the health workforce, have come together to form some of the largest research collaborations in the world, providing high-quality evidence of unjustified variation in practice,^5^ adverse effects of wound infections^3^ and, most recently, to describe the dangerous impact of the COVID pandemic^7^ on surgical care across the world. This has demonstrated the capability of surgical teams, beyond their routine responsibilities, to work together for the benefit of their patients^8 9^ and to quantify the patient benefit of good surgical care.

These studies have been carried out across 100 countries and more.^10^ LMICs have been major contributors. This has provided evidence that is both delivered within the country and because of scale can also provide results that are generalisable. These data, collected in real time (often over a few weeks), are more persuasive for governments and policy makers to use for improved policy and management decisions.

The healthcare system has a sizeable global impact on the carbon footprint and operative and critical care is a major contributor. It is both a high user of energy and highly dependent on reliable energy supplies. It is additionally a major user of single-use products. Ledda and colleagues^11^ ^10^in this issue discuss how by working with hospital managers, surgical/anaesthetic departments can be drivers for the introduction of sustainable energy sources, the reduction in single-use materials and the use of volatile gases. The article also describes the operating theatre as a controlled environment within which the benefits of sustainable practice can be measured.

Health systems have come under overwhelming pressure from the COVID pandemic, from which many are still struggling to recover. While external stressors will differentially affect the health system, surgery has been identified as a highly vulnerable area for early failure. The reasons for this vulnerability and its adverse effect on the health system are explored by Glasbey and colleagues^12^ in this supplement. Once again, the key role of high-quality research into developing resilient surgical systems is identified. They use examples of how such research including the WHO Situational Assessment Tool-13 and Surgical Preparedness Index-9 can support transdisciplinary evaluation of interconnected services, pathways and providers. Findings from such studies will be used to inform on implementation of government and international initiatives.

In the final article in this supplement, Kamarajah and colleagues review the results from a global trial in terms of patient outcomes in first referral hospitals.^13^ These prospective data collected from seven countries on three continents give some encouraging findings concerning the quality of surgical outcomes in these settings and demonstrate the willingness and capability of operative and critical care teams to collect these data.

This supplement, through the four accompanying articles, explores how a resilient surgical service with sufficient capacity can strengthen health systems, drive change for sustainable and resilient working practice and provide resilience when those systems are stressed by (external) pressures. We believe that the global surgical and perioperative care research networks, which historically have not been considered a visible contributor and important player in strengthening health systems, have finally earned the deserved space as a vital stakeholder in strengthening health systems.
